# Hong Kong Adolescents’ Participation in Political Activities: Correlates of Violent Political Participation

**DOI:** 10.1007/s11482-023-10143-6

**Published:** 2023-01-27

**Authors:** Lu Yu, Mingyue Gu, Ko Ling Chan

**Affiliations:** 1 Department of Applied Social Sciences, The Hong Kong Polytechnic University, Hung Hom, Kowloon, Hong Kong; 2 Department of English Language Education, The Education University of Hong Kong, Kowloon, Hong Kong

**Keywords:** Adolescent, Political participation, Violence, Risk and protective factors, Hong Kong

## Abstract

The present study aimed to examine Hong Kong junior secondary school students’ participation in different types of political activities, to identify profiles of adolescents based on their political participation, and to examine potential protective and risk factors associated with adolescents’ violent political participation during the social unrest in Hong Kong from a positive youth development perspective. A total of 2,016 students (age = 13.92 ± 1.10 years) recruited from 24 secondary schools in Hong Kong participated in an online survey six months after the social unrest subsided. The findings provide a comprehensive descriptive profile of Hong Kong adolescents’ political participation. Four clusters of adolescents with homogeneous patterns of political participation were identified: (1) “Politically Inactive” (42.6%); (2) “Legal Participant” (27.5%); (3) “Radical/Violent Activist” (13.0%); and (4) “Peaceful Activist” (17.0%). Logistic regression analysis showed that being female, born in Hong Kong, having a weak local identity and a strong national identity, a high level of bonding, prosocial involvement and prosocial norms, a low level of parental psychological control and family conflict, and a good parent–child relationship were associated with a low risk of adolescents’ violent political participation. The findings point to the needs to further promote social cohesion in Hong Kong society, to help adolescents avoid the potentially essentialized dichotomy in their identity construction, and to develop programs targeting the identified risk and protective factors to prevent adolescents from engaging in political violence and to promote their civic participation.

## Hong Kong Adolescents’ Participation in Political Activities: Correlates of Violent Political Participation

Over the past decade, the social movement in Hong Kong and other developed areas in the world has gradually shifted from peaceful, to contentious, to illegal and radical activities (Cheng, 2016; Uba & Bosi, 2022). In particular, on 12 June 2019, the Hong Kong SAR government’s attempt to introduce the Fugitive Offenders Amendment Bill triggered another mass social movement labelled the Anti-Extradition Law Amendment Bill movement. This event served as the breeding ground for a series of subsequent radical and violent social movements, including but not limited to street blockades, building sieges, arson, physical confrontations with law enforcement officers and citizens with different political viewpoints, and even suicides. These radical behaviours did not fully die out until the end of 2019. Young people, including secondary school students, were the main participants in these radical events.

Although the violent aspects of young people’s protests are now acknowledged within society as almost ubiquitous, there are variations in people’s attitudes towards this behaviour. Some people have argued that these actions cannot be construed as violent at all because the participants had no intention of inflicting harm upon others (Ismangil & Lee, 2021). Others suggest that violence arose not from malice but as an act of self-defence in response to what they considered oppressive political conditions (Lam-Knott, 2017), and should not be categorized as violence in normal terms. Sympathetic attitudes towards violent political action are associated with the process of “radicalization,” which has been defined as an enhanced preparation for and an accentuated engagement with intergroup conflict (McCauley & Moskalenko, 2008). Some scholars have pointed out this worrying trend of radicalism in the work of the leading thinkers in the social movements of Hong Kong (Cheng, 2014). In a recent study (Kennedy et al., 2018), the authors shared similar observations of the radical tendencies of young people in Hong Kong and warned that “current youth disaffection and alienation is well entrenched in anti-government feelings, perhaps to the point where little can be done to reverse it.” Such a rise in student radicalism, and the associated violent political actions of young people, not only presents a challenge to the social order, but also jeopardizes the wellbeing of the participants themselves (Sharp et al., 2014). The wellbeing of young people in general has also been negatively affected. A recent survey by the Hong Kong Federation of Youth Groups (2019) of 2,685 students from 14 secondary schools showed that 41.7% were highly stressed, and 24% felt anxious about the tense social atmosphere.

Despite the worrying situation, evidence on Hong Kong young people’s participation in political violence and its prevention remains limited. Specifically, there have been few systematic studies focused on Hong Kong secondary school students’ participation in these violent and destructive political actions. What are the patterns of Hong Kong students’ political participation? What are the characteristics of those who engaged in political violence? Which factors may prevent youth from participating in violent political actions? Research addressing these gaps has the potential to support the development and implementation of efforts that effectively address adolescent engagement in political violence.

## Radical Political Participation: An Ecological Model-Based Positive Youth Developmental Approach

While young people’s participation in illegal/violent political actions has been conceptualized in different ways (Ekman & Amnå, 2012), the violent nature of the behaviour represents significant short- and long-term risks for their development. We therefore propose that the ecological model-based positive youth development (PYD) approach offers a unique lens through which to understand and address this issue (e.g., Damon, 2004; Lerner et al., 2006). According to this perspective, there are a wide range of protective factors (i.e., developmental assets) at different ecological levels including individual, family, school, community to the wider society that can promote young people’s healthy development, help them reach their full potential, and prevent youth risk behaviors (Damon, 2004). With reference to how these developmental assets might reduce risk behaviors, several potential mechanisms have been proposed. First, positive assets buffer the negative influence of risk factors on adolescents. An example given by Catalano et al. (2004) was that individual with a clear and positive identity may be less likely to engage in risk behaviors caused by peer pressure. Second, developmental assets may compensate the harmful consequences of risk behaviors (Schwartz et al., 2007). For instance, children with good family relationship might still get addicted to the Internet but with fewer harmful effects. Third, the accumulation of multiple positive assets can lead to reduced risk behavior (Yu & Shek, 2021). Fourth, specific assets have molecular protection against certain risk behaviors due to the characteristics of the assets (Kim et al., 1998). It has been reported that social skills and engagement with school reduce young people’s involvement in substance use.

Based on this positive youth developmental approach, young people who have participated in violent political activities also have developmental assets within themselves and in various ecological systems (Benson, 2003) that may enable them to reduce their involvement in violence and contribute to society in a more constructive way. Studies have shown that there is a consistent and positive relationship between young people’s illegal/radical political actions and their participation in legal political activities, the latter being regarded as a desirable developmental outcome (Dahl & Stattin, 2016). This suggests that the two forms of political participation may share some common antecedents, while at certain points there are factors that divert young people along different paths. In one longitudinal study, social responsibility positively predicted adolescents’ readiness for participation in legal protest and negatively predicted readiness to engage in violent political action (Schmid, 2012). If these factors are identified, adolescents’ participation in violent political action may well be prevented.

A number of empirical studies have supported the protective effects of developmental assets in the prevention of various interrelated risk behaviours (Catalano et al., 2004). However, their role in relation to participation in political violence has rarely been examined. Nevertheless, the literature on violence and political behaviour in young people suggests that at individual, family, school, and community levels (Melendez-Torres et al., 2016)—there are factors that may play an important role in preventing young people from engaging in violent activity.

At individual level, positive youth development attributes such as prosocial values, social- emotional competence, and problem-solving skills all serve as important protective factors. They not only directly steer young people away from violent behaviours but also offset the potential harmful effects of other risk factors (e.g., poverty; Shek et al., 2019). Studies on youth radicalization have also found that personal uncertainty leads to radical beliefs and behaviours when there is perceived injustice and intergroup threat, suggesting the protective effect of having a clear and positive identity in the radicalization process (Verkuyten, 2018). However, there is a lack of direct evidence on the influence and relative importance of different positive youth development attributes on youth participation in violent political activities.

At the family level, a positive parent–child relationship, high level of family functioning, consistent and authoritative parenting behaviours, and parents taking an interest in the child’s school life are all associated with reduced likelihood of violent behaviour among young people (Matjasko et al., 2012). In contrast, family dysfunction, parental psychological control, and conflict have been linked with youth radicalization (Campelo et al., 2018).

At the school level, positive connections with teachers and the school, academic success, and interacting with prosocial and nonviolent peers all decrease violent behaviour, while negative perceptions of teachers and challenges to authority are related to adolescents’ illegal political participation and radicalism (Johnson, 2009). Researchers reported that adolescents’ approval of political violence was associated with their reluctance to accept authority (Dahl & Stattin, 2016).

With particular reference to illegal political participation by young people in Hong Kong, several factors unique to this setting have been identified. A strong local identity, a weak national identity, and indiscriminate hostility toward mainland China all increase the risk of illegal political participation and radicalization (Wong et al., 2019). Civic knowledge, understanding China based on experiential learning, discussion of different socio-political issues as part of family interaction, and collective views that violence is unacceptable at school are all linked with reduced intention to engage in illegal political action (Zhu et al., 2020). Such unique factors have not been systematically examined in empirical studies.

## The Present Study

In the present study, the first objective was to provide a descriptive profile of Hong Kong junior secondary school students’ participation in different types of political activities during the social unrest that occurred between June 2019 and January 2020. Secondly, we aimed to use cluster analysis to develop a typology to identify profiles of adolescents based on their political participation. Categorizing students in terms of their political activities will help us to better understand the current landscape of Hong Kong youths’ political participation, understand differences among people of different categories, and help predict how they are going to act in future. Thirdly, we aimed to identify potential protective and risk factors that are associated with students’ risk of being involved in illegal and violent political activities. These factors would be examined at the individual, family, and school levels. Factors specific to Hong Kong, i.e., local and national identity, and perceptions about China, would also be investigated.

## Methods

### Sampling

The target population was Hong Kong Grade 7 to Grade 9 students. A two-stage stratified cluster sample design was adopted. In Stage 1, two identifiers were used to stratify the sample: (1) financing mode of the school (i.e., government schools, aided schools, direct subsidy scheme/private schools); and (2) location within the major regions of Hong Kong (Hong Kong Island, Kowloon, and New Territories). These factors were considered as influential in schools’ contribution to students’ political attitudes and participation. Appendix Table 7 shows the number of schools in each of the nine strata. As the majority were aided schools (*N* = 362, 71.68%), four institutions were randomly selected from each region for aided schools; for government and direct subsidy scheme/private schools, two were randomly selected from each region. As a result, a total of 24 schools from the nine strata were selected to participate. In Stage 2, students studying in Grade 7 to 9 from each sampled school were invited to complete an online survey. Fifty students from each grade were invited to participate based on convenience sampling.

### Participants and Procedure

Data collection was conducted in June 2020. Due to the COVID-19, secondary schools in Hong Kong were suspended for most of the period between February and October 2020. Collective activities held by people outside of the schools were not allowed. This meant we were unable to conduct the survey in classroom settings. Instead, our research assistants waited outside the selected schools and invited students to participate in the study after the end of the school day. Students were briefed on the purpose of the study and provided with a QR code to access the online survey. If they agreed to participate, they would call their parents while still on site to seek their approval. The study purpose and explanation of data handling were repeated to parents and students, stressing the guarantee of confidentiality and anonymity. After both parents and students had signed an online consent form, students proceeded to complete the online questionnaire, which took about 20 min. Each participant was provided with a 100hkd voucher after completion of the survey as an incentive.

Of 3,032 students the research team approached, 2,426 students (80.0%) agreed to participate in the study, and 2,016 students (997 boys and 1,019 girls) (83.1% of the initially recruited participants), including 669 Grade 7 students, 672 Grade 8 students, and 675 Grade 9 students from the 24 selected schools eventually completed the online survey, which means an overall response rate of 66.5%. Students’ age ranged between 12 and 17 years, with a mean age of 13.92 (SD = 1.10). The majority were born in Hong Kong (N = 1,889, 93.7%), 5.8% (N = 117) in mainland China, and 10 students in other places. Table 1 further summarizes the demographic characteristics of the participants.

**Table 1:** Demographic Characteristics of Participants (N = 2,016)

Variable	Frequency	Percentage (%)
Gender		
Male	997	49.45
Female	1,019	50.55
No. of years living in mainland China		
Never	1,758	87.20
Less than one year	63	3.13
One to five years	173	8.58
Five years or longer	22	1.09
Father’s highest education level		
Primary school or below	45	2.23
Secondary school	1,098	54.46
College or Polytechnic (non-degree courses)	175	8.68
University or above (bachelor’s or above)	309	15.33
Don’t know	389	19.30
Mother’s highest education level		
Primary school or below	35	1.74
Secondary school	1170	58.04
College or Polytechnic (non-degree courses)	151	7.49
University or above (bachelor or above)	282	13.99
Don’t know	378	18.75

Given the sensitivity of the survey content, the issue of non-responses should be addressed. Because we did not record reasons why some students withdrew from the study and have no data of those non-responded students for comparison with those who have completed, another method was adopted to examine whether the participants of the present study were different from the general secondary school student population. We further compared the demographic characteristics of Secondary One (Grade 7) participants of the current study with another group of Secondary One students who joined a different study on Internet addiction at about the same time. The results showed that there is no significant difference in the proportion of non-local students and gender ratio between the two samples; more students reported from intact families in the present study (N = 589, 88%) than those participated in the Internet addiction study (N = 1,172, 78.9%), *x*^*2*^ = 25.71, *p* < 0.001. In addition, Secondary One participants of the present study (age = 12.79 ± 0.61 years) were younger than the Secondary One participants of the Internet addiction study (age = 13.19 ± 0.54 years), *F* = 64.92, p < 0.001. These findings provide some evidence for the representativeness of the participants of the present study.

### Measures

The online questionnaire consisted of a combination of items developed by the research team and validated instruments with good psychometric properties, which are explained below. Before we administered the online survey (in Chinese), a pilot study had been conducted with five secondary school students based on a convenient sampling. The students all expressed that they had no difficulties in understanding the items and were able to respond without difficulties.

#### Students’ Participation in Different Types of Political Actions

Items related to a variety of legal and illegal political actions were either excerpted from published scales or formulated by the researchers based on observed political behaviours among young people over the past two years in Hong Kong. A panel consisted of a Professor of youth violence and victimization, a senior researcher of political science, and the first author with expertise in youth development identified a total of 30 items and further categorized them into four types based on the local socio-political context of Hong Kong: (1) legal and conventional activities (seven items), such as “signing a petition;” (2) legal and unconventional activities (seven items), such as “participating in a school boycott;” (3) non-violent/destructive illegal actions (four items), such as “writing political messages on the wall to express political views;” (4) violent/destructive illegal actions (11 items), such as “participating in political activities involving damage to public property;” plus one general item “breaking the law for political reasons”. Students were asked to indicate their frequency of participation in each activity on a four-point rating scale: 1 = never, 2 = rarely, 3 = sometimes, 4 = very often. Cronbach’s alphas for the four subscales are 0.93 (legal conventional political activity), 0.95 (legal unconventional political activity), 0.91 (illegal non-violent political activity), and 0.96 (illegal violent political activity) based on the current sample of students.

In addition, participants were asked to indicate their attitudes towards the 30 political activities based on a five-point rating scale: 1 = I certainly do NOT approve; 2 = I tend to NOT approve; 3 = Neither approve nor disapprove; 4 = I tend to approve; and 5 = I certainly approve. The mean scores of the four subscales are calculated to indicate participants’ attitudes towards different types of political activities. Cronbach’s alphas for the four subscales measuring attitudes are 0.91 (legal conventional political activity), 0.93 (legal unconventional political activity), 0.92 (illegal non-violent political activity), and 0.97 (illegal violent political activity) based on the current sample of students.

#### Individual Factors

##### Positive Youth Development Attributes

The validated Chinese Positive Youth Development Scale (CPYDS) was used to measure students’ 15 Positive Youth Development attributes (Shek et al., 2007), namely bonding, resilience, emotional competence, social competence, cognitive competence, behavioural competence, moral competence, self-determination, belief in the future, clear and positive identity, prosocial norms, spirituality, prosocial involvement, and recognition for positive behaviour (three items for each of these); and self-efficacy (two items). The 15 positive youth development attributes were first proposed by Catalano et al. (2004) based on a synthesis of key constructs identified from effective positive youth development programs in the United States. The CPYDS has been validated in a few empirical studies based on large samples of Chinese populations. Items were rated using a six-point Likert-type scale ranging from 1 = strongly disagree to 6 = strongly agree. Cronbach’s alpha for the subscales based on the current sample was all above 0.70.

##### Social Responsibility

We adopted an existing three-item scale that has been used in several large-scale surveys (Schmid, 2012) to measure social responsibility. The scale asks respondents to rate, using a five-point scale (1 = not important at all and 5 = very important) how important it is to them “to be considerate to others,” “to take responsibility for others,” and “to help other people.” The Cronbach’s alpha for the scale in the present study was 0.82.

##### Perceptions about Mainland China, Local Identity, and National Identity

To capture students’ perceptions about mainland China, a six-item scale previously used in a public policy research project (Yu et al., 2020) was adopted. The six items capture students’ perceptions of mainland China in terms of its economic development, culture, quality of life, relationship with Hong Kong, and Chinese people (Cronbach’s α = 0.90). Two items were used to measure students’ local and national identity. Respondents indicated the extent to which they would agree with the statements “I am a Hong Konger,” and “I am Chinese” using a seven-point Likert-type scale: 1 = strongly disagree, 4 = neutral, and 7 = strongly agree.

#### Family Factors

##### Parenting Behaviours

To measure parenting behaviours, the validated Parent–Child Subsystem Quality Scale (Shek & Law, 2016) was adopted. The scale consists of 17 items in three dimensions: (1) parental behavioural control (7 items) (Cronbach’s α = 0.79); (2) parental psychological control (4 items) (Cronbach’s α = 0.87); and (3) the quality of the parent–child relationship (3 items) (Cronbach’s α = 0.80). Students responded to each item using a four-point Likert-type scale. For each dimension, students’ average score across the subscale items was calculated.

##### Family Functioning

The 9-item Chinese Family Assessment Instrument (Siu & Shek, 2005) was used to assess family mutuality (3 items), communication (3 items), and family conflict (3 items). For each dimension, students’ average score across the subscale items was calculated. The Cronbach’s αs for the three subscales were 0.73 (family mutuality), 0.73 (family communication), and 0.66 (family conflict).

##### Family Discussion of Socio-Political Issues

Four items were used to assess how frequently students had the opportunity to freely discuss socio-political issues with their parents. Students rated the frequency of each item using a five-point scale: 1 = never, 2 = rarely, 3 = sometimes, 4 = often, and 5 = always (Cronbach’s α = 0.90). Students’ average score across the four items indicated the level of family discussion of socio-political issues, with a high score representing more frequent discussion. An example item is “In my family, we talk about our perspectives on current socio-political events”.

#### School Factors

##### Perceptions of Teachers

Five items were used to ask students about their perceptions of their teachers (e.g., “Most teachers don’t like me”). Responses were collected using a four-point Likert scale (Cronbach’s α = 0.83). Students’ average score across the five items represents their overall perception of teachers. The higher the score, the more negative the students’ perception.

##### Discussion on Socio-Political Issues and Collective Views against Violence at School

Six items were designed to assess how frequently students were provided with opportunities to freely discuss socio-political issues at school. Students rated the frequency of each item using a five-point scale from 1 = never, 3 = sometimes, to 5 = always (Cronbach’s α = 0.90). High scores representing more frequent discussions. Schools’ collective views against violence was rated with one item “In our school, we are taught to be against all forms of violence” using the same rating scale as described above.

### Data Analysis

We firstly computed descriptive statistics of the participants’ engagement in four major forms of political activities. Secondly, to understand the disparity in students’ political participation, clusters of participation in different political activities among the 2,016 cases were identified based on the SPSS Two Step Clustering algorithm. Data were analysed using SPSS 26.0. Students’ self-reported participation in the 30 named political activities was recoded into dichotomous variables, with 0 = never participated (i.e., never) and 1 = participated (rarely, sometimes, or very often). The number of clusters was determined a priori based on the two basic dimensions used to differentiate different modes of political participation: conventional vs. unconventional and legal vs. illegal. Based on these dimensions, political activity participants can be naturally categorized into three groups: legal and conventional participants; legal and unconventional participants; and illegal and unconventional participants, as all illegal political participation can be regarded as unconventional. Taking into account another group of people who rarely participate in any political activity, we assume that adolescents can be classified into four clusters. One-way MANOVA was performed to validate the clusters.

To understand what factors might differentiate students in the cluster of violent participants from those in the other clusters, a logistic regression analysis was conducted. Students’ membership of the four clusters was re-coded into a dichotomous variable with 1 = “violent” (i.e., members of the radical/violent activist cluster) and 0 = “nonviolent” (i.e., everyone else). The new variable was then used as the dependent variable in the logistic regression. For the independent variables, students’ demographic characteristics, including gender (1 = male; 0 = female), age, parental education level, place of birth (1 = local; 0 = nonlocal), and years of living in mainland China were entered at the first block; students’ local identity (as a Hong Konger), national identity (as Chinese), and perceptions of mainland China were entered in the second block; and the hypothesized predictors at individual, family, and school levels were all entered at the third block. Multi-collinearity analysis was performed, and the results showed that the Variance Inflation Factor (VIF) values for all predictors ranged between 1.183 and 2.326, indicating an acceptable level of correlations among the predicators.

## Results

### Participation of Hong Kong Secondary Students in Relation to Different Types of Political Activities

Table 2 shows the frequency of the students in the sample that had participated in different types of political activities between June 2019 and January 2020. Firstly, with regards to participation in legal and conventional activities, 50.8% of the respondents reported that they had “forwarded political messages on social media,” around 46% had “signed a petition (either online or offline),” and more than 30% of students had “distributed leaflets with political content” (32.6%) or “created or joined an online group for politics or public affairs” (30.7%). The activity in which they participated least frequently was “contacting a legislator or government official for public affairs in person or by phone/email,” with 18.11% of the students reporting that they had ever done so.

**Table 2:** Participation of Hong Kong Junior Secondary School Students in Different Types of Political Activities (N = 2,016)

	Never		Rarely		Sometimes		Very Often		Yes	
Legal and conventional	N	%	N	%	N	%	N	%	N	%
1. Sign a petition (either online or offline)	1084	53.77	424	21.03	421	20.88	87	4.32	932	46.23
2. Distribute leaflets with a political content	1359	67.41	344	17.06	246	12.20	67	3.32	657	32.59
3. Forward political messages in social media	991	49.16	443	21.97	397	19.69	185	9.18	1025	50.84
4. Participate in activities organized by political parties or organizations	1422	70.54	308	15.28	221	10.96	65	3.22	594	29.46
5. Contact a legislator or government official for public affairs in person or by phone/email	1651	81.89	203	10.07	124	6.15	38	1.88	365	18.11
6. Donate or raise money for political affairs	1454	72.12	326	16.17	195	9.67	41	2.03	562	27.88
7. Create or join any online group for politics or public affairs	1398	69.35	292	14.48	255	12.65	71	3.52	618	30.65
Legal and unconventional	N	%	N	%	N	%	N	%	N	%
1. Take part in a peaceful demonstration or political rally	961	47.67	478	23.71	439	21.78	138	6.85	1055	52.33
2. Participate in a school boycott	1350	66.96	253	12.55	316	15.67	97	4.81	666	33.04
3. Wear a specific badge or T-shirt to express political views	926	45.93	387	19.20	503	24.95	200	9.92	1090	54.07
4. Boycott any product for political reasons	895	44.39	334	16.57	505	25.05	282	13.99	1121	55.61
5. Buy products for political reasons	912	45.24	337	16.72	500	24.80	267	13.24	1104	54.76
6. Form human chains to express political views	1212	60.12	329	16.32	344	17.06	131	6.50	804	39.88
7. Gather in housing estates or shopping malls to sing and/or shout political slogans	1256	62.30	360	17.86	300	14.88	100	4.96	760	37.70
Illegal and nonviolent	N	%	N	%	N	%	N	%	N	%
1. Participate in illegal political gatherings, actions, or occupation	1477	73.26	308	15.28	191	9.47	40	1.98	539	26.74
2. Jump over turnstiles at MTR stations to express political views	1613	80.01	263	13.05	116	5.75	24	1.19	403	19.99
3. Call upon others to participate in unauthorized assemblies or demonstrations	1615	80.11	264	13.10	110	5.46	27	1.34	401	19.89
4. Write or post political messages on the wall to express political views	1442	71.53	271	13.44	253	12.55	50	2.48	574	28.47
Illegal and violent	N	%	N	%	N	%	N	%	N	%
1. Stage a protest by blocking the road	1734	86.01	190	9.42	72	3.57	20	0.99	282	13.99
2. Set fires on the street or inside buildings	1845	91.52	126	6.25	32	1.59	13	0.64	171	8.48
3. Participate in political activities involving damage to public property (e.g., MTR, traffic lights)	1852	91.87	121	6.00	29	1.44	14	0.69	164	8.13
4. Occupy public buildings as an act of protest	1782	88.39	150	7.44	65	3.22	19	0.94	234	11.61
5. Participate in vandalizing politically opposed shops	1845	91.52	122	6.05	42	2.08	7	0.35	171	8.48
6. Participate in political activities involving violent confrontations with the police or pro-police groups	1784	88.49	171	8.48	47	2.33	14	0.69	232	11.51
7. Participate in political activities involving violent confrontations with anti-police groups	1805	89.53	153	7.59	45	2.23	13	0.64	211	10.47
8. Doxing political opponents	1781	88.34	175	8.68	42	2.08	18	0.89	235	11.66
9. Verbally abuse/insult the police or political opponents	1740	86.31	191	9.47	62	3.08	23	1.14	276	13.69
10. Have physical confrontations with political opponents	1837	91.12	121	6.00	45	2.23	13	0.64	179	8.88
11. Isolate or bully political opponents and/or their family members at school	1815	90.03	141	6.99	48	2.38	12	0.60	201	9.97

Yes = students who had participated at least once (Rarely + Sometimes + Very Often)

Secondly, students reported higher levels of participation in legal but unconventional rather than conventional actions. More than half of the respondents reported that they had “taken part in a peaceful demonstration or political rally” (52.3%), “boycotted or bought products for political reasons” (55.6% and 54.8% respectively), and/or “worn a specific badge or T-shirt to express political views” (54.1%). Furthermore, around one third of the students reported that over the past year they had “participated in a school boycott” (33%), or “gathered in housing estates or shopping malls to sing and/or shout political slogans” (37.7%) to express political opinions. This seems to suggest that Hong Kong secondary school students were more interested in using unconventional ways to engage in socio-political issues.

For students’ participation in illegal but nonviolent political activities, although the proportion of students who had participated in these activities was lower than for legal activities, 20% to 30% of the respondents reported that they had “written or posted political messages on the wall to express political views” (28.5%), “participated in illegal political gatherings or occupation” (26.7%), or “jumped over turnstiles at MTR stations to express political views” (20%). These findings indicate that quite a number of Hong Kong adolescents had engaged in illegal political activities although they had not been violent.

A small proportion of students reported that they had participated in illegal and violent political activities such as “starting fires on the street or inside buildings” (8.5%), “participating in political activities involving damage to public property” (8.1%), or “having physical confrontations with political opponents” (8.9%). However, the same was not true for certain other illegal and violent activities. For example, around 14% of respondents had participated in “staging a protest by blocking the road” and/or “verbally abusing/insulting the police or political opponents.” More than 10% had engaged in “occupying public buildings as an act of protest” (11.6%), “participating in political activities involving violent confrontations with the police, pro-police, or anti-police groups” (11.5% and 10.5% respectively), and/or “doxing political opponents” (11.7%). As participation in illegal and violent political activities could result in serious consequences for young people, more attention needs to be paid to this group.

### Disparities in Hong Kong Secondary School Students’ Political Participation and Associated Factors

#### Patterns of Political Participation

Four distinct clusters with homogenous patterns of political participation were identified in the two-step clustering analysis referred to above. Of the 2,016 participants, 42.6% (*n* = 858) were classified as cluster 1, 27.5% (*n* = 554) as cluster 2, 13.0% (*n* = 262) as cluster 3, and 17.0% (*n* = 342) as cluster 4. Table 3 shows the means and standard deviations of participation in different types of political activities for each cluster. To validate the clusters, two one-way MANOVA analyses were conducted. Students in the four clusters were compared in terms of their reported participation in the 30 political activities included in the survey over the past year, as well as their attitudes towards the four types of political participation (i.e., legal and conventional, legal and unconventional, illegal and non-violent, and illegal and violent). For the first analysis, a statistically significant MANOVA effect was obtained: *F* (90, 5935) = 126.82, *p* < 0.001, *Wilks’ Λ* = 0.04. Follow-up univariate ANOVAs showed that the engagement scores for all political activities were statistically significantly different for students in the four clusters (Table 3). For the second analysis, similar results were generated: F (24, 5815) = 123.37, p < 0.001, Wilk’s lambda = 0.30. The univariate ANOVAs suggest the four clusters of students showed different attitudes toward different types of political activities; the attitudes are consistent with their participation (Table 3).

**Table 3:** Means and standard deviations of students’ participation in different types of political activities and their attitudes by cluster (*N* = 2,016)

Item	Cluster 1 (n = 858)		Cluster 2 (n = 554)		Cluster 3 (n = 262)		Cluster 4 (n = 342)		MANOVA		
Participation	*M*	*SD*	*M*	*SD*	*M*	*SD*	*M*	*SD*	F	*η*^*2*^_*p*_	Pairwise comparisons
1. Sign a petition (either online or offline)	1.15	.42	1.69	.87	2.71	.87	2.66	.61	631.01^***^	.49	C3 & C4 > C2 > C1
2. Distribute leaflets with a political content	1.03	.21	1.18	.48	2.51	.90	2.51	.69	1080.80^***^	.62	C3 & C4 > C2 > C1
3. Forward political messages in social media	1.18	.48	2.03	1.03	2.79	.91	2.74	.77	499.32^***^	.43	C3 & C4 > C2 > C1
4. Participate in activities organized by political parties or organizations	1.01	.11	1.14	.42	2.52	.89	2.34	.82	970.13^***^	.59	C3 > C4 > C2 > C1
5. Contact a legislator or government official for public affairs in person or by phone/email	1.00	.07	1.03	.20	2.00	.96	1.82	.91	408.15^***^	.38	C3 > C4 > C2 & C1
6. Donate or raise money for political affairs	1.01	.12	1.14	.40	2.34	.90	2.17	.80	764.57^***^	.53	C3 > C4 > C2 > C1
7. Create or join any online group for politics or public affairs	1.01	.08	1.18	.46	2.64	.94	2.40	.77	1057.38^***^	.61	C3 > C4 > C2 > C1
8. Take part in a peaceful demonstration or political rally	1.12	.36	1.90	.78	3.06	.77	2.83	.66	1034.13^***^	.61	C3 > C4 > C2 > C1
9. Participate in a school boycott	1.01	.10	1.34	.65	2.59	1.03	2.64	.80	873.57^***^	.57	C3 & C4 > C2 > C1
10. Wear a specific badge or T-shirt to express political views	1.09	.36	2.24	.87	3.16	.78	2.93	.68	1104.96^***^	.62	C3 > C4 > C2 > C1
11. Boycott any product for political reasons	1.06	.32	2.49	.87	3.27	.76	3.08	.66	1412.70^***^	.68	C3 > C4 > C2 > C1
12. Buy products for political reasons	1.03	.20	2.46	.86	3.26	.75	3.08	.66	1568.91^***^	.70	C3 > C4 > C2 > C1
13. Form human chains to express political views	1.00	.08	1.53	.75	2.97	.81	2.75	.72	1234.16^***^	.65	C3 > C4 > C2 > C1
14. Gather in housing estates or shopping malls to sing and/or shout political slogans	1.00	.05	1.46	.71	2.88	.85	2.49	.75	1030.62^***^	.61	C3 > C4 > C2 > C1
15. Participate in illegal political gatherings, actions, or occupation	1.00	.03	1.14	.38	2.60	.79	1.91	.81	920.32^***^	.58	C3 > C4 > C2 > C1
16. Jump over turnstiles at MTR stations to express political views	1.00	.03	1.10	.31	2.27	.83	1.52	.75	573.12^***^	.46	C3 > C4 > C2 > C1
17. Call upon others to participate in unauthorized assemblies or demonstrations	1.00	.00	1.10	.32	2.36	.79	1.46	.70	695.90^***^	.51	C3 > C4 > C2 > C1
18. Write or post political messages on the wall to express political views	1.00	.06	1.27	.55	2.67	.79	1.99	.92	729.93^***^	.52	C3 > C4 > C2 > C1
19. Stage a protest by blocking the road	1.00	.06	1.06	.26	2.13	.85	1.17	.49	589.79^***^	.47	C3 > C4 > C2 > C1
20. Set fires on the street or inside buildings	1.00	.05	1.05	.21	1.75	.86	1.02	.13	366.24^***^	.35	C3 > C4 & C2 & C1
21. Participate in political activities involving damage to public property (e.g., MTR, traffic lights)	1.00	.00	1.04	.19	1.74	.86	1.02	.15	362.46^***^	.35	C3 > C4 & C2 & C1
22. Occupy public buildings as an act of protest	1.00	.03	1.05	.24	2.11	.89	1.05	.22	717.94^***^	.52	C3 > C4 & C2 & C1
23. Participate in vandalizing politically opposed shops	1.00	.03	1.03	.19	1.77	.81	1.02	.13	446.52^***^	.40	C3 > C4 & C2 & C1
24. Participate in political activities involving violent confrontations with the police or pro-police groups	1.01	.11	1.06	.25	1.98	.82	1.03	.18	604.83^***^	.47	C3 > C2 > C1; C3 > C4
25. Participate in political activities involving violent confrontations with anti-police groups	1.00	.08	1.05	.22	1.94	.83	1.02	.13	594.76^***^	.47	C3 > C4 & C2 & C1
26. Doxing political opponents	1.00	.03	1.05	.23	2.00	.84	1.06	.26	603.64^***^	.47	C3 > C4 & C2 > C1
27. Verbally abusing/insulting the police or political opponents	1.00	.05	1.13	.43	2.09	.81	1.08	.38	511.84^***^	.43	C3 > C4 & C2 > C1
28. Having physical confrontations with political opponents	1.00	.07	1.03	.19	1.87	.86	1.01	.08	505.58^***^	.43	C3 > C4 & C2 & C1
29.Isolating or bullying political opponents and/or their family members at school	1.00	.00	1.05	.24	1.92	.82	1.02	.16	572.06^***^	.46	C3 > C4 & C2 & C1
30. Breaking the law for political reasons	1.00	.00	1.02	.17	1.75	.79	1.03	.22	435.51^***^	.39	C3 > C4 & C2 & C1
Attitude											
1. Attitude towards legal and conventional political actions	2.64	.59	3.01	.43	3.30	.46	3.27	.39	203.42***	.23	C3 & C4 > C2 > C1
2. Attitude towards legal and unconventional political actions	2.47	.66	3.01	.49	3.41	.49	3.41	.42	340.15***	.34	C3 & C4 > C2 > C1
3. Attitude towards illegal and non-violent political actions	1.92	.68	2.21	.69	2.93	.67	2.74	.66	214.90***	.24	C3 > C4 > C2 > C1
4. Attitude towards illegal and violent political actions	1.61	.59	1.77	.61	2.51	.79	2.03	.75	139.05***	.17	C3 > C4 > C2 > C1

^*^p < .05, ^**^p < *.*01, ^***^ *p* < *.*001

Bonferroni post-hoc comparison tests showed that cluster 1 had statistically significantly lower mean scores than the other three clusters for all the given political activities except for “contacting a legislator or government official for public affairs” (*p* = 0.73; non-significant difference between cluster 1 and 2). Students in cluster 3 had statistically higher levels of participation in all types of political activities than those in cluster 1 (*p* < 0.001) and cluster 2 (*p* < 0.001). With a few exceptions for legal conventional political participation (e.g., “signing a petition,” “distributing leaflets with political content,” and “participating in a school boycott”), cluster 3 also reported higher levels of participation than did cluster 4. Cluster 4 students reported more participation in all types but violent political activities than both cluster 1 (p < 0.001) and cluster 2 (p < 0.001); for violent political participation, cluster 4 scored lower than cluster 3. The MANOVA results therefore provide evidence supporting the validity of the cluster analysis.

#### Profile of the Clusters

**Fig. 1:**
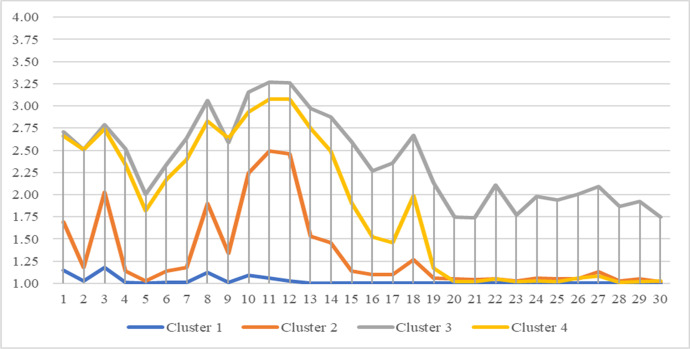
Students’ Participation in 30 Political Activities by Cluster (N = 2,016). *Note.* Items 1–7 = legal and conventional political activities; items 8–14 = legal and unconventional political activities; items 15–18 = illegal nonviolent political activities; items 19–29 = illegal violent political activities; item 30 = an overall item on illegal political activities

The characteristics of the four clusters are described further below. Figure 1 illustrates the mean score for each cluster in terms of participation in different political activities, and Table 4 summarizes the percentages of students in each cluster who participated in such activities. The first cluster (*n* = 858, 42.6%) reported the lowest level of participation of the four clusters in all 30 political activities, legal and illegal. A small percentage of students in this cluster reported that they had participated in legal political activities: e.g., 14.2% had “forwarded political messages on social media”, and 11.3% had “taken part in a peaceful demonstration.” For other legal political activities, the proportion of participants ranged from 0.2% (“gathering in a housing estate to shout political slogans”) to 7.3% (“wearing a specific T-shirt to express political views”). The majority (but not all) of the students in this group had never participated in any illegal activities. Therefore, this cluster may best be described as “Politically Inactive.”

**Table 4:** Proportion of Students Who Had Participated in Different Types of Political Activities by Cluster (*N* = 2,016)

Item	Cluster 1 (*n* = 858)	Cluster 2 (*n* = 554)	Cluster 3 (*n* = 262)	Cluster 4 (n = 342)
1. Sign a petition (either online or offline)	12.4%	45.7%	90.8%	98.0%
2. Distribute leaflets with a political content	2.2%	14.4%	86.7%	96.8%
3. Forward political messages in social media	14.2%	58.5%	93.5%	97.7%
4. Participate in activities organized by political parties or organizations	1.2%	11.7%	86.6%	85.4%
5. Contact a legislator or government official for public affairs in person or by phone/email	0.5%	3.1%	63.0%	52.3%
6. Donate or raise money for political affairs	1.2%	12.3%	80.9%	79.5%
7. Create or join any online group for politics or public affairs	0.6%	15.3%	87.0%	87.7%
8. Take part in a peaceful demonstration or political rally	11.3%	65.7%	98.1%	98.5%
9. Participate in a school boycott	0.7%	25.5%	79.4%	90.9%
10. Wear a specific badge or T-shirt to express political views	7.3%	77.8%	98.1%	99.1%
11. Boycott any product for political reasons	4.3%	87.9%	98.5%	99.1%
12. Buy products for political reasons	2.6%	87.4%	98.5%	99.4%
13. Form human chains to express political views	0.3%	38.6%	96.6%	97.7%
14. Gather in housing estates or shopping malls to sing and/or shout political slogans	0.2%	34.5%	95.4%	92.7%
15. Participate in illegal political gatherings, actions, or occupation	0.1%	13.4%	92.7%	64.6%
16. Jump over turnstiles at MTR stations to express political views	0.1%	9.7%	84.0%	37.4%
17. Call upon others to participate in unauthorized assemblies or demonstrations	0.0%	8.8%	88.9%	34.8%
18. Write or post political messages on the wall to express political views	0.3%	20.9%	93.9%	61.1%
19. Stage a protest by blocking the road	0.3%	6.1%	76.7%	12.9%
20. Set fires on the street or inside buildings	0.2%	4.5%	52.7%	1.8%
21. Participate in political activities involving damage to public property (e.g., MTR, traffic lights)	0.0%	3.6%	51.9%	2.3%
22. Occupy public buildings as an act of protest	0.1%	4.3%	73.3%	5.0%
23. Participate in vandalizing politically opposed shops	0.1%	3.1%	56.1%	1.8%
24. Participate in political activities involving violent confrontations with the police or pro-police groups	0.3%	6.0%	70.6%	3.2%
25. Participate in political activities involving violent confrontations with anti-police groups	0.2%	4.7%	67.6%	1.8%
26. Doxing political opponents	0.1%	5.2%	28.6%	5.3%
27. Verbally abuse/insult the police or political opponents	0.2%	9.9%	77.1%	5.0%
28. Have physical confrontations with political opponents	0.1%	3.2%	60.3%	0.6%
29. Isolate or bully political opponents and/or their family members at school	0.0%	4.0%	66.0%	1.8%
30. Break the law for political reasons	0.0%	1.8%	57.3%	2.3%

The second cluster (*n* = 554, 27.5%) reported moderate levels of participation in legal political activities, particularly nonconventional ones. For example, 87.9% of students reported that they had “boycotted a product for political reasons,” while 87.4% had “bought products for political reasons”, and 77.8% had “worn a specific badge express political views.” An interesting finding is that these students reported low participation in legal and conventional political activities, with “forwarding political messages in social media” being the most frequent such activity (58.5%), followed by “signing a petition” (45.7%). Other legal and conventional political activities were participated in by 15% or less of the students in this cluster. Cluster 2 students also had significantly lower levels of participation in all types of political activities than clusters 3 and 4 but participated more in legal protest than the Politically Inactive cluster. Therefore, cluster 2 may best be described as “Legal Participants.”

The third cluster was the smallest identified in the analysis, with 262 respondents (13.0%). This group is characterized by its members’ high level of participation in all four types of political activities. In particular, this group reported the highest participation in illegal political activities (both nonviolent and violent) of all four groups. For instance, more than 90% reported that they had “participated in illegal political gatherings or occupation” and/or “written political messages on the wall to express political views”. For violent political activities, 76.7% of students in this group had “staged a protest by blocking the road,” 73.3% “occupied public buildings as an act of protest,” 70.6% “participated in political activities involving violent confrontations with the police or pro-police groups, and 77.1% “verbally abused/insulted the police or political opponents.” This cluster is described as “Radical/Violent Activist.”

The fourth and final cluster consisted of 342 students (17.0%). This group was characterized by a high level of participation in legal political activities, a moderate level of participation in illegal but nonviolent activities, and a low level of participation in violent political activities. Their participation in legal political activities was comparable to that of the Radical/Violent Activist group (cluster 3), and significantly higher than the Politically Inactive and Legal Participant clusters. Specifically, all seven legal and unconventional political activities were participated in by more than 90% of students in this group. For example, nearly all of them had “boycotted” (99.1%) or “bought products” (99.4%) for political reasons, “worn a specific badge or T-shirt to express political views” (99.1%), or “taken part in a peaceful demonstration or political rally” (98.5%). In terms of illegal but nonviolent political activities, cluster 4 students scored lower than the Radical/Violent Activist group but higher than the Politically Inactive and Legal Participant clusters. For violent political activities, cluster 4 students’ participation was comparable to that of the Legal Participant cluster, lower than the Radical/Violent Activist cluster, and higher than the Politically Inactive student group. This cluster can be described as “Peaceful/Nonviolent Activist.”

#### Characteristics of the Radical/Violent Activist Cluster

Among the four clusters, the Radical/Violent Activist group of students is of most concern. To understand what factors differentiate them from the other groups of participants, a logistic regression analysis was performed. The results are summarized in Table 5. Firstly, at the demographic level, being born in Hong Kong was associated with a decreased risk of participating in violent political activities (*B* = -0.76, *p* < 0.05, *OR* = 0.47), while being male (*B* = 0.50, *p* < 0.001, *OR* = 1.65), of an older age (*B* = 0.14, *p* < 0.05, *OR* = 1.15), and having a higher parental education level (*B* = 0.16, *p* < 0.05, *OR* = 1.17) were associated with an increased risk. In particular, the risk of students born in Hong Kong participating in violent political activities was about half of the risk for students born elsewhere. Moreover, the likelihood of violent political participation increased when holding a local identity (*B* = 0.28, *p* < 0.01, *OR* = 1.32) and decreased when having a national identity (*B* = -0.43, *p* < 0.001, *OR* = 0.65).

**Table 5:** Results of the Logistic Regression Analysis Predicting Membership of the Radical/Violent Activist Cluster (N = 2,016)

	*Model 1*						*Model 2*							*Model 3*				
	*B*	*SE*	*Wald*	*OR*	*95% CI for OR*		*B*	*SE*	*Wald*	*OR*	*95% CI for OR*			*B*	*SE*	*Wald*	*OR*	*95% CI for OR*
					*Lower*	*Upper*					*Lower*	*Upper*					*Lower*	*Upper*
Block 1																		
Gender ^a^	0.50^***^	0.15	12.06	1.65	1.25	2.20	0.55^***^	0.15	13.34	1.73	1.29	2.32	0.43^*^	0.17	6.57	1.53	1.11	2.12
Age	0.14^*^	0.07	4.62	1.15	1.01	1.31	0.10	0.07	2.06	1.10	0.97	1.26	-0.06	0.08	0.61	0.94	0.81	1.10
Parental educational level	0.16^*^	0.08	3.96	1.17	1.00	1.37	0.11	0.08	1.79	1.12	0.95	1.31	0.08	0.09	0.72	1.08	0.90	1.30
Place of birth ^b^	-0.76^*^	0.34	5.01	0.47	0.24	0.91	-0.93^**^	0.35	7.02	0.40	0.20	0.79	-1.27^**^	0.41	9.69	0.28	0.13	0.63
Previous living experience in China	-0.15	0.28	0.30	0.86	0.50	1.48	0.04	0.28	0.03	1.05	0.60	1.82	-0.25	0.32	0.63	0.78	0.41	1.45
Block 2																		
Local identity							0.28^**^	0.09	10.74	1.32	1.12	1.56	0.22^*^	0.10	5.27	1.25	1.03	1.52
National identity							-0.43^***^	0.06	58.41	0.65	0.58	0.73	-0.46^***^	0.07	47.08	0.63	0.55	0.72
Perceptions about China							0.17	0.14	1.55	1.19	0.91	1.56	0.40	0.16	6.18	1.49	1.09	2.04
Block 3																		
Bonding													-0.41^*^	0.17	5.98	0.66	0.48	0.92
Resilience													0.20	0.17	1.48	1.22	0.89	1.69
Social competence													-0.12	0.16	0.52	0.89	0.65	1.22
Recognition of positive behavior													-0.16	0.16	1.01	0.86	0.63	1.16
Emotional competence													0.01	0.16	0.00	1.01	0.74	1.38
Cognitive competence													0.21	0.17	1.61	1.24	0.89	1.71
Behavioral competence													0.05	0.17	0.10	1.05	0.76	1.47
Moral competence													0.05	0.17	0.09	1.05	0.75	1.48
Self-determination													0.10	0.17	0.31	1.10	0.79	1.53
Self-efficacy													0.38^*^	0.15	5.99	1.46	1.08	1.97
Clear and positive identity													0.27	0.17	2.59	1.31	0.94	1.81
Beliefs in the future													0.18	0.17	1.09	1.19	0.86	1.65
Prosocial involvement													-0.33^*^	0.16	4.07	0.72	0.53	0.99
Prosocial norms													-0.57^**^	0.17	10.63	0.57	0.40	0.80
Social responsibility													0.13	0.14	0.91	1.14	0.87	1.49
Spirituality													0.24^*^	0.10	5.44	1.27	1.04	1.54
Parental behavioral control													-0.07	0.25	0.07	0.94	0.57	1.52
Parental psychological control													0.43^**^	0.15	8.32	1.54	1.15	2.06
Parent–child relationship													-0.93^***^	0.19	23.36	0.40	0.27	0.58
Family mutuality													-0.08	0.21	0.14	0.93	0.62	1.39
Family conflict													0.34^**^	0.13	7.02	1.40	1.09	1.80
Family communication													0.05	0.18	0.07	1.05	0.74	1.48
Opportunities to discuss socio-political issues at home													-0.06	0.13	0.19	0.94	0.73	1.22
Negative teacher behaviors													-0.31	0.18	2.88	0.74	0.52	1.05
Opportunities to discuss socio-political issues at school													0.87^***^	0.16	27.77	2.38	1.72	3.28
School’s collective views against violence													-0.20	0.12	3.02	0.82	0.65	1.03
χ^2^					28.26^***^						119.77^***^						318.08^***^	
Nagelkerke R^2^						.03						.13						.31

^a^ Gender is set for males compared to females. ^b^ Place of birth is set for local students compared to nonlocal students

^*^p < .05, ^**^p < .01, ^***^ *p* < .001. OR = Odds Ratio

In terms of positive youth development attributes, bonding (*B* = -0.41, *p* < 0.05, *OR* = 0.66), prosocial norms (*B* = -0.57, *p* < 0.01, *OR* = 0.57) and prosocial involvement (*B* = -0.33, *p* < 0.05, *OR* = 0.72) were all associated with a decreased risk of violent political participation, whereas self-efficacy (*B* = 0.38, *p* < 0.05, *OR* = 1.46) and spirituality (*B* = 0.24, *p* < 0.05, *OR* = 1.27) were associated with an increased risk. At the family level, parental psychological control (*B* = 0.43, *p* < 0.01, *OR* = 1.54) and family conflict (*B* = 0.34, *p* < 0.01, *OR* = 1.40) increased the likelihood of a student participating in violent political activities, while having a good parent–child relationship decreased it (*B* = -0.93, *p* < 0.001, *OR* = 0.40). At the school level, the only significant factor was having opportunities to discuss socio-political issues at school (*B* = 0.87, *p* < 0.001, *OR* = 2.38). Increased incidence of discussions was associated with a significantly heightened risk of students taking part in violent political protests. To provide further information on the effect size, we performed a separate logistic regression in which all predictors were standardized scores of the variables. The results are shown in Table 6.

**Table 6:** Results of the Logistic Regression Analysis Predicting Membership of the Radical/Violent Activist Cluster (N = 2,016) with predictors being standardized

	*Model 1*						*Model 2*								*Model 3*						
	*B*	*SE*	*Wald*	*OR*	*95% CI for OR*		*B*	*SE*	*Wald*		*OR*		*95% CI for OR*		*B*	*SE*		*Wald*	*OR*	*95% CI for OR*	
					*Lower*	*Upper*							*Lower*	*Upper*						*Lower*	*Upper*
Block 1																					
Gender ^a^	.25***	.07	12.06	1.29	1.12	1.48	.27***	.08	13.34		1.32		1.14	1.52	.21*	.08	6.57		1.24	1.05	1.46
Age	.15*	.07	4.62	1.17	1.01	1.34	.11	.07	2.06		1.11		.96	1.29	-.07	.09	.61		.94	.79	1.11
Parental educational level	.14*	.07	3.96	1.15	1.00	1.31	.09	.07	1.79		1.10		.96	1.26	.07	.08	.72		1.07	.91	1.26
Place of birth ^b^	-.19*	.08	5.01	.83	.71	.98	-.23**	.09	7.01		.80		.68	.94	-.31**	.10	9.69		.73	.60	.89
Previous living experience in China	-.05	.09	.30	.95	.79	1.14	.01	.09	.02		1.01		.84	1.22	-.08	.11	.63		.92	.74	1.13
Block 2																					
Local identity							.26**	.08	10.74	1.30			1.11	1.52	.21*	.09	5.27		1.23	1.03	1.48
National identity							-.63***	.08	58.41	.53			.45	.63	-.68***	.10	47.08		.51	.42	.62
Perceptions about China							.11	.09	1.55	1.12			.94	1.33	.25*	.10	6.18		1.29	1.06	1.58
Block 3																					
Bonding															-.25*	.10	5.98		.78	.63	.95
Resilience															.13	.11	1.48		1.14	.92	1.41
Social competence															-.07	.10	.52		.93	.76	1.14
Recognition of positive behavior															-.11	.11	1.01		.90	.73	1.11
Emotional competence															.01	.10	0		1.01	.82	1.23
Cognitive competence															.14	.11	1.61		1.15	.93	1.43
Behavioral competence															.03	.11	.10		1.03	.84	1.28
Moral competence															.03	.11	.09		1.03	.83	1.29
Self-determination															.06	.11		.31	1.06	.85	1.33
Self-efficacy															.26*	.11		5.99	1.30	1.05	1.60
Clear and positive identity															.18	.11		2.59	1.20	.96	1.49
Beliefs in the future															.12	.11		1.09	1.12	.90	1.40
Prosocial involvement															-.23*	.11		4.07	.79	.63	.99
Prosocial norms															-.38**	.12		10.62	.69	.55	.86
Social responsibility															.09	.09		.91	1.09	.91	1.30
Spirituality															.20*	.09		5.44	1.22	1.03	1.44
Parental behavioral control															-.03	.11		.07	.97	.79	1.20
Parental psychological control															.29**	.10		8.32	1.33	1.10	1.62
Parent–child relationship															-.50***	.10		23.36	.61	.50	.74
Family mutuality															-.04	.11		.14	.96	.78	1.19
Family conflict															.23**	.09		7.02	1.26	1.06	1.50
Family communication															.03	.11		.06	1.03	.83	1.27
Opportunities to discuss socio-political issues at home															-.04	.10		.19	.96	.79	1.16
Negative teacher behaviors															-.15	.09		2.88	.86	.72	1.02
Opportunities to discuss socio-political issues at school															.63***	.12		27.77	1.88	1.48	2.37
School’s collective views against violence															-.20	.12		3.02	.82	.65	1.03
χ^2^						28.26^***^						119.77^***^								318.08^***^	
Nagelkerke R^2^						.03								.13							.31

^a^ Gender is set for males compared to females. ^b^ Place of birth is set for local students compared to nonlocal students

^*^p < .05, ^**^p < .01, ^***^ *p* < .001. OR = Odds Ratio

## Discussion

The present study is the first that provides a descriptive profile of Hong Kong secondary school students’ participation in different types of political activities during the social unrest that took place between 2019 and 2020 in Hong Kong, based on a large sample. The findings suggest that Hong Kong students’ participation in political activities can be clustered into four distinct groups: the Politically Inactive Group, the Legal Participant Group, the Peaceful Activist Group, and the Radical/Violent Activist group. Specifically, 13% of the students were classified as Radical/Violent Activist who reported the highest level of participation in illegal and violent political activities across all four groups. Characteristics of this group of adolescents at individual, family, and school levels were identified that will bear important theoretical and practical implications in preventing youth violent political participation.

In general, we found that Hong Kong adolescents were not receptive to participation in legal and conventional political activities. Except for “forwarding political messages on social media” and “signing a petition”, 70% of students reported that they never participated in any other conventional political activities. In contrast, more students had engaged in legal and unconventional political actions. Besides, over 20% of students reported having participated in illegal and non-violent political activities. This is consistent with previous findings showing that young people tend to voice grievances more often in non-institutionalized forms of political activities than institutionalized ones (Rainsford, 2017; Weiss, 2020). Some researchers have explained that young people’s preferences for unconventional forms of political participation is associated with the fact that they are provided with less opportunities to participate in institutionalized activities (Grasso, 2016).

In the context of Hong Kong, recent studies suggest that youth have become increasingly active in non-institutionalized forms of political participation (Weiss, 2020) and they have a “latent preparedness” (Reichert, 2021) to become politically active when they are dissatisfied with the government and perceive themselves as being responsible to act against unjust laws. Reichert’s (2021) study, based on university students, showed that the more students were involved in either online or offline political activities, the less satisfied they were with the government in Hong Kong. As Shek (2020) pointed out, young people in Hong Kong have lost faith in many facets of the government over the policy delivery concerning their current and future welfare, which resulted in adolescents pinning their hope on non-conventional political activities that they believed to be more influential in leading to a fundamental change of the government’s stances on an array of political and livelihood issues. Our finding provides support to the notion that Hong Kong youth possess a “latent preparedness” and are ready for political participation when needed.

While the majority of students reported zero or rare violent political participation, 13% of respondents had engaged in violent political activities (i.e., the Radical/Violent Activist group). A few characteristics of this group were identified. Firstly, at the demographic level, four factors were found to be associated with a high risk of participating in violent political activities. Compared to the other groups, Radical/Violent Activists were more likely to be older, male, born outside of Hong Kong, and have relatively higher parental educational levels. The effects of age and gender are consistent with previous findings in which older adolescents were found to be more likely to translate their political attitude and opinions into action (Quintelier & Hooghe, 2012); boys prefer more confrontational forms of political action (e.g., blocking traffic), whereas girls tend to engage more in social action-related behaviours (e.g., voting or contributing signatures for a cause) (Hooghe & Stolle, 2004).

The finding that Hong Kong immigrant youth, especially mainland China-to-Hong Kong immigrants (91.5% of all students who were “born outside Hong Kong,”) had a higher risk of violent political participation, seems to be contradicting with existing findings. Past literature has shown that mainland Chinese immigrants in Hong Kong reported relatively lower levels of political participation; they were more likely to approve of the political and economic status quo, and less likely to vote for pro-democracy opposition parties, as compared to Hong Kong local citizens (e.g., Policy 21 Limited, 2013). The present finding seems to be contradicting with the previous research. One possible explanation could be the objective deprivation and relative deprivation theory (Brush, 1996; Atkinson et al., 2002). There is evidence showing that immigrants have been facing serious difficulties in adjusting to life in Hong Kong, due to such things as unacceptable housing conditions, low income, social isolation, and discrimination (Wong et al., 2012). The deprivation—both the feeling and the reality of social exclusion deriving from their affiliation to a social marginal group and poverty—experienced by immigrant youths, could lead to a trend of embracing violent behaviours and of identifying with radical political attitudes (Mudde, 2010). In the present study, we did find that parental highest educational level, as an indicator of family economic status, was lower for the mainland China-born immigrant students than for the local students. Further analysis showed that there is no interactive effect between immigrant status and parental educational level on youth violent political participation. This seems to suggest that on the one hand, the deprived nature of immigrants may contribute to adolescents’ violent political participation, and on the other hand, being born in mainland China itself may also predict youth participation in radical political activities. These findings suggest the need to further promote social cohesion and integration in Hong Kong society. Effective measures should be taken to address the difficulties encountered by immigrant families. Meanwhile, policymakers should consider how to improve young immigrants’ sense of belongingness and empower them to participate in legal socio-political activities.

Unexpectedly, “higher parental educational level” and “frequent family discussion of socio-political issues” were found to be associated with a high risk of adolescents’ violent political participation. The literature suggests that youth with higher socio-economic status are more likely to be politically engaged and have regular political discussions at home, while lower socio-economic status engenders lower levels of social trust, which further leads to violent political engagement (Weiss, 2020). One possible explanation for the present finding is that some well-educated parents are sceptical of the government and supportive of anti-government political actions, including illegal and violent ones. Parental political opinions can influence their adolescent children in many ways, such as through family discussion of socio-political issues. Research findings have shown that people from different social classes joined in the protests, including professionals and the middle-class population (Shek, 2020), and that more educated people in Hong Kong were more supportive of the protests (Sing, 2020). The present study, however, did not measure parents’ political attitude directly. Future research should collect data on both parental and children’s political participation so as to arrive at a more rigorous explanation of the relationship between family economic status and adolescents’ violent political participation.

Another notable finding is that the Radical/Violent Activist group was characterized by a strong local identity (being a Hong Konger) and a weak national identity (being Chinese). Previous research has revealed a national identity crisis among a significant plurality of the Hong Kong population, especially the younger generation (Veg, 2017). Students’ negative perceptions of mainland China and its socio-political system are one of the many factors that accounted for their resulting endorsement of the local, indigenous Hong Konger identity and for their disavowal of the national Chinese identity. Adolescents’ prioritization of the local identity over the national identity in the identity confirmation process implies their lost sense of attachment to mainland China and their emphasis on the profound distinctiveness of the different facets of Hong Kong society, such as its independent judiciary, bilingualism, and education system vis-à-vis the mainland (Morris & Vickers, 2015). Consistent with the present findings, Chow et al. (2020) reported that one’s probability of engaging in political activities increases by means of his/her identification as a “Hong Konger” and association with the “Hong Kong identity.” These findings highlight the importance of helping Hong Kong adolescents to develop a sense of belonging to mainland China and thus establish a civic national identity as Chinese. Previous research supports that direct service-learning experiences in mainland China and cultural and recreation activities that help students to understand the history of the country are effective ways to strengthen Hong Kong university students’ national identity (Yu et al., 2020). It is recommended that such constructive approaches can also be adopted in the civic education curriculum in secondary school settings.

A few positive youth development attributes were found to be associated with lower risks of youth violent political participation, including a sense of bonding, identification with prosocial norms, and prosocial behaviours. Many studies have examined the relationship between social bonds and delinquent behaviour (Chan & Chui, 2013). Positive social bonding to family and school and belief in the moral order have been found to inhibit deviant and violent behaviours (Catalano & Hawkins, 1996). Unsatisfied needs for social bonding in early childhood could increase the emotional attraction of groups (such as gangs) with violent and authoritarian values and leadership (Pedersen, 2004). The present findings move existing research forward by showing the negative relationship between healthy social bonding and adolescents’ violent political participation. To prevent youth from engaging in violent political activities, educators and policymakers may provide more opportunities for adolescents to develop positive relationships and social bonding with healthy adults and peer groups embracing prosocial beliefs such as altruism and volunteerism, as well as for them to practice more helping, caring, and cooperating behaviours in the community. There are research findings which show that school-wide programs that provide diverse opportunities for adolescents to engage in voluntary work and community service can effectively promote students’ development of prosocial attributes and positive values (Shek, 2019). These programs may also have the potential to prevent youth from engaging in violent political actions, while contributing to society in a constructive way.

On the other hand, students of the Radical/Violent group also reported a stronger sense of self-efficacy and a higher level of spirituality than the other groups. In the literature of positive youth development, self-efficacy and spirituality have both been considered important developmental assets (Benson, 2003; Catalano et al., 2004). Empirical studies also show that the two attributes can lessen violent behaviours (Darawashy & Haj-Yahia, 2018; Ichikawa et al., 2017) and enhance positive outcomes among young people (Masten, 2013). With regards to the present finding, we speculate that it is violent political participation that enhances adolescents’ self-efficacy and spirituality, rather than vice versa. According to social cognitive theory, youth can gain their self-efficacy from past successful experiences (Bandura, 1993). Participating in these violent political activities may boost adolescents’ confidence and belief in their ability to exert control over their own lives and social environment, i.e., self-efficacy; it may also provide adolescents with a sense of meaning for their lives or spirituality. Nonetheless, based on the current cross-sectional study, we cannot exclude the possibility that self-efficacy may play a role in youths’ violent political participation, as there has been research showing that individuals with higher levels of self-efficacy are more likely to act out their political opinions and commit political violence than those with lower levels of self-efficacy (Schlegel, 2020). The causal relationship between violent political participation and the two positive youth development attributes should be further examined in longitudinal research.

With regards to family factors, Radical/Violent political participants reported higher levels of parental psychological control, more family conflict, and worse parent–child relationships than students in other groups did. The findings converge with a large variety of studies showing that negative parenting practices and family dysfunction are associated with adolescents’ aggression (Smokowski et al., 2015), antisocial behaviour (Steinberg, 2000), and involvement in violence (Tolan et al., 2003). In particular, adolescents’ self-perceived parental psychological control has been consistently related to more emotional and behavioural problems. Parents’ psychological control was also found to weaken children’s self-control, which in turn makes adolescents vulnerable to behavioural problems.

While the present findings yield evidence for a possible role of positive parenting and family functioning in preventing adolescents’ violent political participation, we cannot rule out the possibility that youths’ participation in violent political actions created more family conflict and worsened parent–child relationships, especially when parents disapprove of their adolescent children’s political participation or hold different political stances. In fact, several studies have reported increased intra-familial conflict and different levels of renegotiation in family relationships of active participants during the social unrest in Hong Kong (Ho et al., 2018; Lo et al., 2021). Lower relationship quality and lower congruence in family members’ political attitudes predicted more serious subsequent family conflicts (Chan et al., 2018), which can lead to or worsen the existing mental health burden of adolescents and their family members. The present finding highlights the need for and importance of providing extra support to families with adolescents, especially those who had participated in the social unrest. Education for parents of adolescents at the secondary school stage needs to be further promoted to equip them with sufficient parenting knowledge and skills. Specific guidance could be provided for parents on how to communicate constructively and hold constructive discussions with their adolescent children on socio-political issues to build up mutual understanding, and how to handle conflicts.

At the school level, the only factor that differentiated the Radical/Violent group from the other groups was the “opportunities to discuss socio-political issues at school”. Contrary to our expectation that students with more civic knowledge would be less likely to get involved in violent political action, Radical/Violent Activists reported more encouragement and more opportunities provided by teachers to discuss different views about social-political issues at school than students in other groups did. This finding seems to be consistent with Lee’s earlier (2016) study, which found that in Hong Kong, Liberal Studies and teachers are two critical factors contributing to the political socialization at schools of young protest participants. Having peers who discuss important political controversies and joining political activist peer groups were also found to be two common ways for Hong Kong adolescents to become active social movement participants. As a matter of fact, recent studies did suggest that youth political actions and protest were supported by many Hong Kong citizens (Kwong, 2016) and there was no strong public condemnation of young people’s violent political behaviours (such as vandalism or use of physical force to settle interpersonal conflicts). When these behaviours and activities are viewed and discussed as acceptable or even necessary means to get justice under certain circumstances (e.g., fight against police brutality), it is no wonder that the more frequent the discussions at school or at home, the more likely it is that the adolescents who joined the discussions would show violent political actions.

The current finding also echoes Shek (2020) and colleagues’ (2018) observation that systematic and coordinated civic and national education has been absent in Hong Kong. Even among teachers, there is a lack of consensus regarding what constitutes “good citizenship.” On one hand, interviews with young radical political participants showed that they identified teachers as the most important factor for them to develop their active democratic citizenship (Lee, 2016). On the other hand, a study with Hong Kong primary school teachers revealed that teachers perceived “protest participation” as “the least important characteristic of good citizenship” and “the least effective means for cultivating good citizenship” (Wong, 2018, p. 107). These findings clearly suggest that there is a discrepancy among teachers themselves in terms of their understanding about civic engagement and good citizenship, which points to the need of enhancing systematic civic education for both teachers and students. Policymakers should critically review the current curriculum and teaching practices for liberal studies and civic education. A more comprehensive civic and national education curriculum that incorporates a broad and clear framework of “active citizenship” accompanied with important components of “law-abiding awareness,” “holistic youth development,” and “character education” is critically needed. Training and guidelines for teachers should also be provided to empower them to build a positive school culture, maintain supportive relationships with students, and effectively promote students’ discussion on socio-political issues.

A note of caution in interpreting the present findings is warranted. First, the findings are based on students’ self-reported data and are correlational in nature. Although our findings may indicate factors at individual, family, and school levels could increase or decrease the risk of adolescents participating in violent political actions, the findings can be interpreted in both ways. In particular, the outcome variables were measured based on recalled experiences. Although most of the predictors were either with constant value (e.g., gender, birthplace, parental educational level) or relatively stable (e.g., parenting style, bonding, prosocial norms), some factors are likely to change over months, especially during adolescence. For example, it is possible that adolescents’ self-efficacy can be enhanced by their involvement in violent political actions. Only longitudinal data may provide more insight into the problem of causality and tease apart competing causal hypotheses. The cross-sectional design also prevents us from excluding the influence of other variables on the observed relationships between youth violent political participation and the identified factors. For instance, having a peer group with radical political orientation may increase both the frequency of discussion of socio-political issues at school and students’ political violence (Lee, 2016). Future study should take into account the effect of peer influence and other potential third factors (e.g., parents’ political participation) in explaining youths’ participation in different types of political activities.

Another related limitation is that we did not dig much into the mechanism underlying the identified relationships. For example, the processes involved in the positive relationship between spirituality and youth violent political participation are not entirely clear. Possible mediators such as one’s meaning in life were not investigated. The positive association between “opportunities to discuss socio-political issues at school” and youth’s involvement in violent actions may be moderated by teachers’ approval/disapproval of violent political participation. In fact, in the literature with reference to the underpinning process of how positive youth development attributes may reduce violent behaviors, yet a comprehensive theory is lacking. The potential pathway(s) from positive attributes to the reduction of violent behaviors need further investigation. In future studies, more attention should be paid to the potential mediators and moderators to elaborate the theoretical mechanism.

Secondly, although the participating schools were randomly selected from all schools based on the financial modes in Hong Kong, students were invited to participate in the study on a voluntary basis. It is possible that students who had participated in violent activities were reluctant to report their behaviours or participate in the study. Therefore, selection bias and non-responses issue may exist. Besides, all data were collected from self-reported online questionnaire, and social desirability could have inadvertently influenced the quality of data. A few measures were taken to reduce the selection bias and the influence of non-responses issue. First, we collected the data after social unrest subsided and social stability was restored when the national security law had not been implemented. Second, we adopted anonymous online survey to provide a sense of security for the participants to answer the questions. Third, our team members clearly described the purposes of the survey, provided background information about the PI and funding scheme of the project, highlighted the importance of their responses to the survey, and assured the participants that all responses would be kept confidential when they recruited the participants. Fourth, a cash voucher of HKD$100 was provided to each respondent upon completion of the survey. This incentive also encouraged more students to participate in the survey. While we hope these measures may help offset the selection bias and social desirability, future studies should consider recruiting a more representative sample of students and adopting other objective measures or research methods, such as social media analysis, to investigate youth political participation in Hong Kong.

Thirdly, we only compared the Radical/Violent Activist group with the other three groups as a whole because we aimed to understand factors that may differentiate adolescents with violent political participation from those without. It would be meaningful to further investigate the characteristics of the other three groups, for example, the Peaceful Activist group, who were active in both legal and illegal political activities, without engaging in violence and destruction. This group may represent a proportion of adolescents with a high level of civic engagement and social responsibility but who were provided with inadequate knowledge and opportunities for political participation. Further studies should be conducted to understand this group of young people and to explore what new channels can be created to encourage them to effectively express their political opinions and engage them in policymaking.

Fourthly, we adopted a “person-centred approach”, cluster analysis, to group students based on their participation in different types of political activities, while there are criticisms regarding the use of cluster analysis to generate a representation of the underlying structure of the data. In particular, unlike “variable-centred approach” such as factor analysis, cluster analysis does not yield a “best” solution and therefore we cannot guarantee the generated four clusters are a more optimal solution than others. Nonetheless, the number of clusters in the present study was determined a priori based on existing dimensions of political activities, i.e., theory-driven, and our research purpose was to identify groups of individuals demonstrating different patterns of political participation, instead of identifying sets of variables. Therefore, cluster analysis is considered as a more appropriate method for the present study than factor analysis despite of its advantages.

Last but not least, the categorization of political activities into different types (legal vs. illegal; conventional vs. nonconventional; violent vs. non-violent) was based on the local socio-political context in Hong Kong at the time of data collection as well as the judgement by an expert panel. The grouping of the same political actions could be quite different in another socio-cultural context, such as in the U.S., or other Western countries. While researchers could use the items to compare the political participation rate by adolescents cross-culturally; it would also be interesting to examine the difference in how researchers from countries with diverse political systems view or categorize these political activities.

In summary, our findings suggest that although the overall participation rate of Hong Kong secondary school students in political activities was not high, there is a significant proportion of adolescents who were involved in violent political activities. We found that being female, having been born in Hong Kong, having a weak local identity (Hong Konger) and a strong national identity (Chinese), a high level of bonding, prosocial involvement and prosocial norms, a low level of parental psychological control and family conflict, and a good parent–child relationship were associated with a low risk of adolescents’ violent political participation.

## Conclusion

Findings of the present study contribute to our understanding about adolescents’ violent political participation in Hong Kong, a context in which violent extremism has rarely been examined. The identification of potential protective factors at multiple-levels, especially positive youth development attributes and culture specific factors, expands the existing literature on youth political violence. These individual and psychosocial factors may be focused on in the development of prevention and intervention programmes in future. The divisive relationship between local and national identity, as reflected in the protective factors, points to the necessity of developing youth understanding of the compatible nature of the two identities (local and national) through guidance and support at the familial and school levels, which will help avoid the potentially essentialized dichotomy in their identity construction. Meanwhile, further studies based on longitudinal research design must be conducted to clarify the role of self-efficacy, spirituality, and students’ discussion of socio-political issues at home and at school in their participation in different types of political activities.

Essentially, promoting the quality of life and positive development of youth, as well as actively engaging young people in formal political processes may be the key entry points to prevent youth political violence.

## Appendix

**Table 7:** Total Number of Secondary Schools in the Nine Strata and Number of Selected Schools in Each Stratum

Financing mode	HK Island Total (selected)	Kowloon Total (selected)	New Territories Total (selected)	Sum Total (selected)
Government schools	8 (2)	9 (2)	14 (2)	31 (6)
Aided schools	55 (4)	102 (4)	205 (4)	362 (12)
Direct subsidy scheme/ Private schools*	28 (2)	41 (2)	43 (2)	112 (6)
Total	91 (8)	152 (8)	262 (8)	505 (24)

* Excluding schools only offering evening classes or with senior form students
